# The Epidemiology of Multiple Sclerosis in Scotland: Inferences from Hospital Admissions

**DOI:** 10.1371/journal.pone.0014606

**Published:** 2011-01-27

**Authors:** Adam E. Handel, Lynne Jarvis, Ryan McLaughlin, Anastasia Fries, George C. Ebers, Sreeram V. Ramagopalan

**Affiliations:** 1 Wellcome Trust Centre for Human Genetics, University of Oxford, Oxford, United Kingdom; 2 Department of Clinical Neurology, University of Oxford, Oxford, United Kingdom; 3 National Health Service Scotland, Edinburgh, United Kingdom; James Cook University, Australia

## Abstract

**Background:**

Multiple sclerosis (MS) is a neurological disorder with a highly characteristic disease distribution. Prevalence and incidence in general increase with increasing distance from the equator. Similarly the female to male sex ratio increases with increasing latitude. Multiple possible risk factors have been hypothesised for this epidemiological trend, including human leukocyte antigen allele frequencies, ultraviolet exposure and subsequent vitamin D levels, smoking and Epstein-Barr virus. In this study we undertook a study of medical records across Scotland on an NHS health board level of resolution to examine the epidemiology of MS in this region.

**Methods and Results:**

We calculated the number and rate of patient-linked hospital admissions throughout Scotland between 1997 and 2009 from the Scottish Morbidity Records. We used weighted-regression to examine correlations between these measures of MS, and latitude and smoking prevalence. We found a highly significant relationship between MS patient-linked admissions and latitude (r weighted by standard error (r_sw_) = 0.75, p = 0.002). There was no significant relationship between smoking prevalence and MS patient-linked admissions.

**Discussion:**

There is a definite latitudinal effect on MS risk across Scotland, arising primarily from an excess of female MS patients at more Northerly latitudes. Whether this is a true gradient or whether a threshold effect may apply at particular latitude will be revealed only by further research. A number of genetic and environmental factors may underlie this effect.

## Introduction

Multiple sclerosis (MS) is a complex neurological disorder characterised by demyelination and axonal loss.[1] MS shows a marked variation by latitude throughout the world, with increasing prevalence and incidence with increasing distance from the equator.[2], [3] This latitudinal effect has been observed in a large number of different studies in the Northern hemisphere and also holds true in the Southern hemisphere. Much of this latitude gradient appears to stem from an excess of female MS patients at higher latitudes.[4], [5]

Multiple genetic and environmental risk factors have been hypothesised to underlie the distribution of MS, including human leukocyte antigen (HLA) allele frequencies, ultraviolet radiation and vitamin D levels, smoking, and infection with Epstein-Barr virus.[6], [7], [8], [9] It is likely that many of these interact together on a population-wide level to determine MS prevalence and incidence.[8], [10], [11]

The Northern parts of Scotland have previously been shown to have a particularly high prevalence of MS.[12] This appears to be responsible for much of the latitudinal variation in MS across the United Kingdom.[13] However recent data on the prevalence of MS in Scotland is not available. Given suggestions that the latitude gradient in MS is decreasing,[5] we undertook an analysis of MS hospital admissions stratified by NHS health board in Scotland in order to examine epidemiological trends in MS in fine detail.

## Methods

Data on hospital admissions for MS between 1997 and 2009 were analysed using a file of linked hospital admission statistics, built from the Scottish Morbidity Records (SMR01) system. SMR01 records all inpatient and day case discharges from non-obstetric and non-psychiatric specialties in NHS Hospitals in Scotland. Probability matching methods have been used to link together individual hospital records for each patient, thereby creating “linked” patient histories. Up to six diagnoses (1 principal, 5 secondary) are recorded on SMR01 returns. All six diagnoses have been used to select Multiple Sclerosis. The following code was used from the International Statistical Classification of Diseases and Other Health Problems, tenth revision (ICD10): Multiple Sclerosis - G35. These diagnoses are made by the lead physician responsible for each patient's care during the admission.

We show data for the areas of the 14 Scottish Strategic Health Boards. MS indirect admission rates were calculated using observed average annual numbers of MS patients from each area with one or more hospital admissions as the numerators and the expected number of average annual MS admissions from census data as the denominators, before multiplying this by the overall MS admissions rate for Scotland. Using data linkage, we identified each person only once for MS, regardless of how many admissions each person had, and recorded their residence at first known admission for MS. To adjust for differences in the age structure of different areas, age-standardisation was undertaken using the indirect method and the age-specific rates in the Scottish population. All rates are expressed per 100 000 population with 95% lower and upper confidence limits. Rates were calculated separately for males and females as well as both sexes combined. We also calculated rates for other autoimmune diseases (rheumatoid arthritis, ulcerative colitis, Crohn's disease and type 1 diabetes mellitus) and a neurological disease (motor neuron disease).

Smoking data was obtained from the Atlas of Tobacco Smoking in Scotland, produced by the NHS Health Scotland, ISD Scotland and ASH Scotland.[14] Population smoking prevalence estimates were used from 2003/2004 and were based upon all age groups combined. Latitudes for each NHS health board were defined as the latitude of the administrative centre for that health board. As anonymised data were used we followed the ethical principles of existing UK data protection legislation and guidance, including two National Statistics (NS) Protocols on Data Access and Confidentiality, and Data Matching and so specific ethical approval was not required for this study.

We performed Pearson and linear weighted regression to compare the admissions for MS, latitude and smoking using MATLAB R2009a. Least-squared weighted regression was conducted based both on standard error estimates and a weighting value derived from NHS health board population numbers. χ^2^ tests were used to compare observed and expected hospital admissions throughout Scottish health boards.

## Results

### MS admissions by NHS health board

The overall number of patients admitted with MS (as an absolute value over the 13 year period and a yearly average) and indirect rate per 100,000 population are shown in **table 1**. Overall 11,094 individual patients were admitted with MS in Scotland between 1997 and 2009. This was equivalent to a yearly average of 853 and an indirect rate of 16.87 per 100,000 population. However, it was clear that the admission rates varied considerably between NHS health boards, with the highest rate in NHS Orkney Islands (37.38 [95% CI 25.40–49.37]) and the lowest in NHS Forth Valley (12.54 [95% CI 5.60–19.47]).

**Table 1:** Overall admissions data for MS in Scotland between 1997 and 2009.

NHS board of residence	Observed patients (13 year total)	Expected patients (13 year total)	Average yearly observed patients	Average yearly expected patients	Indirect rate per 100,000	Lower 95% CI	Upper 95% CI
NHS Ayrshire & Arran	949	816.20	73.00	62.78	19.62	10.94	28.30
NHS Borders	202	246.32	15.54	18.95	13.84	6.55	21.13
NHS Dumfries & Galloway	341	336.33	26.23	25.87	17.11	9.00	25.22
NHS Fife	793	769.55	61.00	59.20	17.39	9.22	25.56
NHS Forth Valley	455	612.48	35.00	47.11	12.54	5.60	19.47
NHS Grampian	1,377	1,153.98	105.92	88.77	20.14	11.34	28.93
NHS Greater Glasgow & Clyde	2,190	2,567.77	168.46	197.52	14.39	6.96	21.83
NHS Highland	900	682.03	69.23	52.46	22.27	13.02	31.52
NHS Lanarkshire	1,051	1,206.56	80.85	92.81	14.70	7.18	22.21
NHS Lothian	1,611	1,688.84	123.92	129.91	16.10	8.23	23.96
NHS Orkney Islands	97	43.78	7.46	3.37	37.38	25.40	49.37
NHS Shetland Islands	72	47.70	5.54	3.67	25.47	15.58	35.36
NHS Tayside	844	851.46	64.92	65.50	16.73	8.71	24.74
NHS Western Isles	73	59.02	5.62	4.54	20.87	11.92	29.83
**Scotland**	**11,094**		**853**		**16.87**		

The data for male and female patients admitted with MS are shown in **tables 2** **and** **3**. The indirect rate for MS in females across Scotland was significantly higher than in males (males 10.69 vs. females 22.64, χ^2^ =  4.28, d.f. = 1, p = 0.04).

**Table 2:** Male only admissions data for MS in Scotland between 1997 and 2009.

NHS board of residence	Observed patients (13 year total)	Expected patients (13 year total)	Average yearly observed patients	Average yearly expected patients	Indirect rate per 100,000	Lower 95% CI	Upper 95% CI
NHS Ayrshire & Arran	304	248.12	23.38	19.09	13.10	6.01	20.19
NHS Borders	56	76.82	4.31	5.91	7.79	2.32	13.27
NHS Dumfries & Galloway	97	104.87	7.46	8.07	9.89	3.73	16.05
NHS Fife	244	235.52	18.77	18.12	11.08	4.55	17.60
NHS Forth Valley	128	187.04	9.85	14.39	7.32	2.02	12.62
NHS Grampian	393	363.14	30.23	27.93	11.57	4.90	18.24
NHS Greater Glasgow & Clyde	691	768.40	53.15	59.11	9.62	3.54	15.69
NHS Highland	280	214.20	21.54	16.48	13.98	6.65	21.30
NHS Lanarkshire	314	363.61	24.15	27.97	9.23	3.28	15.19
NHS Lothian	503	510.87	38.69	39.30	10.53	4.17	16.89
NHS Orkney Islands	27	13.93	2.08	1.07	20.72	11.80	29.64
NHS Shetland Islands	23	15.43	1.77	1.19	15.94	8.11	23.76
NHS Tayside	246	261.03	18.92	20.08	10.08	3.85	16.30
NHS Western Isles	22	19.01	1.69	1.46	12.38	5.48	19.27
**Scotland**	**3,384**		**260.31**		**10.69**		

**Table 3:** Female only admissions data for MS in Scotland between 1997 and 2009.

NHS board of residence	Observed patients (13 year total)	Expected patients (13 year total)	Average yearly observed patients	Average yearly expected patients	Indirect rate per 100,000^1^	Lower 95% CI	Upper 95% CI
NHS Ayrshire & Arran	645	571.39	49.62	43.95	25.55	15.64	35.46
NHS Borders	146	168.92	11.23	12.99	19.56	10.90	28.23
NHS Dumfries & Galloway	244	231.09	18.77	17.78	23.90	14.32	33.48
NHS Fife	551	534.60	42.38	41.12	23.33	13.86	32.80
NHS Forth Valley	328	426.61	25.23	32.82	17.40	9.23	25.58
NHS Grampian	984	778.81	75.69	59.91	28.60	18.12	39.08
NHS Greater Glasgow & Clyde	1,501	1,812.62	115.46	139.43	18.74	10.26	27.23
NHS Highland	620	463.55	47.69	35.66	30.27	19.49	41.06
NHS Lanarkshire	738	848.58	56.77	65.28	19.69	10.99	28.38
NHS Lothian	1,110	1,180.19	85.38	90.78	21.29	12.25	30.33
NHS Orkney Islands	70	29.41	5.38	2.26	53.88	39.49	68.27
NHS Shetland Islands	49	31.32	3.77	2.41	35.42	23.75	47.08
NHS Tayside	598	591.78	46.00	45.52	22.87	13.50	32.25
NHS Western Isles	51	39.14	3.92	3.01	29.50	18.85	40.14
**Scotland**	**7,718**		**594**		**22.64**		

### Correlation of MS admissions with latitude

**Figure 1:**
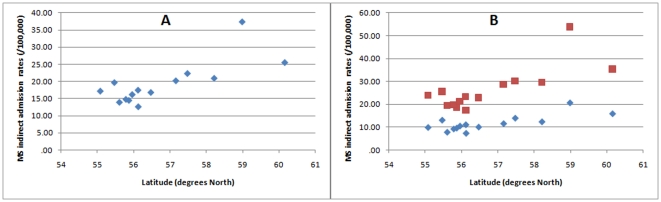
Scatter plots of MS admission rates against latitude. (A) Overall data. (B) Male (blue) and female (red) specific data.

Overall MS hospital admissions showed a strong correlation with latitude (**figure 1a**; r = 0.76, p = 0.001; r weighted by SE (r_sw_) = 0.75, p = 0.002; r weighted by population (r_pw_) = 0.71, p = 0.004). This held true when analysed separately by gender, although the male data was not significant when weighted by population (**figure 1b**; males – r = 0.77, p = 0.001; r_sw_ = 0.74, p = 0.002; r_pw_ = 0.52, p = 0.06; females – r = 0.77, p = 0.001; r_sw_ = 0.76, p = 0.002; r_pw_ = 0.76, p = 0.001).

### Correlation of MS admissions with smoking prevalence

**Figure 2:**
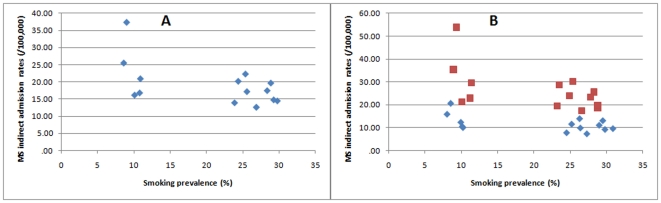
Scatter plots of MS admission rates against smoking prevalence. (A) Overall data. (B) Male (blue) and female (red) specific data.

There was a nominally significant *inverse* correlation with smoking but this was lost in the weighted regression (**figure 2a**; overall – r = −0.55, p = 0.04, r_sw_ = −0.49, p = 0.08; r_pw_ = −0.25, p = 0.39; **figure 2b** **;** males – r = −0.52, p = 0.06, r_sw_ = −0.47, p = 0.09; r_pw_ = −0.21, p = 0.47; females – r = −0.56, p = 0.04; r_sw_ = −0.49, p = 0.08; r_pw_ = −0.25, p = 0.38).

### Sex ratio

**Figure 3:**
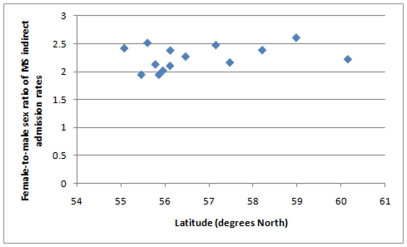
Scatter plots of female-to-male sex-ratio of MS admission rates against latitude.

There was no significant correlation between the sex ratio and latitude in MS patient-linked admissions except when weighted by population (**figure 3**; r = 0.32, p = 0.27; r_sw_ = 0.41, p = 0.15, r_pw_ = 0.75, p = 0.002). There was no relationship between the sex ratio and smoking prevalence (r = 0.27, p = 0.36; r_sw_ = 0.36, p = 0.21; r_pw_ = 0.14, p = 0.64).

### Other autoimmune and neurological diseases

There was no significant overall correlation between indirect admissions rate and latitude for several autoimmune diseases (data not shown; rheumatoid arthritis – r_sw_ = 0.29, p = 0.39; Crohn's disease – r_sw_ = 0.06, p = 0.81; type 1 diabetes mellitus – r_sw_ = 0.26, p = 0.37) or a neurological disease (motor neuron disease – r_sw_ = 0.26, p = 0.37). Indirect admissions rates for ulcerative colitis did show a nominally significant correlation with latitude (r_sw_ = 0.57, p = 0.03) but this was lost after correction for multiple hypothesis testing.

## Discussion

We present here the most up to date available map of the distribution of MS in Scotland. Our findings have shown that the latitude gradient of MS prevalence and incidence observed still exists in Scotland.[2] As suggested by previous work,[2] much of this latitude gradient is as a result of an increase in female MS risk at increasing latitudes. It must be noted, however, that, although our data does support a latitudinal gradient, the data itself could also argue for a “threshold” effect around 57°N. Until more is understood about the aetiological origin for this effect of latitude on MS prevalence, conclusions regarding this must remain tentative.

Smoking does not appear to underlie this latitudinal effect, especially given the apparent *inverse* correlation of smoking prevalence with latitude. Although there is some suggestion that the sex-ratio in MS admissions may be inversely related to smoking prevalence, this is quite unlikely to be a real effect given that where MS risk is highest (NHS Orkney Islands), smoking prevalence is lowest and so, regardless of the ratio between male and female smoking behaviour, it is difficult to envision how the absence of a susceptibility factor could result in a sex-specific *increase* of disease risk.[15] There are clear limitations in using background population measures of smoking prevalence as a surrogate for exposure in patients with MS. In particular, effects on the level of individual patients will not be detected in this sort of population-based data. Future studies should re-examine this question in relation to MS-specific smoking data.

Limitations of record linkage studies using routinely collected administrative data are well known, and include the facts that the data are limited to hospitalised patients and that information about some variables of potential interest, such as social circumstances and ethnicity, are generally unavailable. It is also worth noting that the sample size of regions primarily driving the latitudinal effect (e.g. the Orkney and Shetland Islands) and relatively small and so this effect will need to be confirmed in future work. The fact that latitudinal effects were not seen for other autoimmune and neurological diseases argues strongly against the gradient we observed being solely due to underlying trends in hospital services or background population admissions rates.

The environmental risk factor that most likely explains the latitudinal effect observed in this study is the effect of ultraviolet irradiation on vitamin D biosynthesis. Vitamin D deficiency has already been associated with risk of MS and controls the expression of HLA-DRB1*1501, the key genetic risk factor in MS.[10], [16] However, it is likely that MS prevalence will be explained only by considering a complex interplay of genetic and environmental risk factors, some of which may not yet have been identified [8].

The latitudinal variation in MS prevalence may have important implications for neurological service provision. For example, although the number of MS-relevant clinics per week correlates well with the total number of admissions as one would expect with the tendency of admissions to follow hospital resources (r = 0.69, p<0.01), these show a trend towards *inverse* correlation with MS indirect admissions rates, probably a better estimate of background disease prevalence (r = −0.52, p = 0.06) [17]. MS disease distribution should be considered when evaluating neurology services in Scotland in the future.

In conclusion, the distribution of MS across Scotland shows nearly three-fold variation, at least as measured by hospitalisation rates. The higher rates in the North than South of Scotland persist; they are consistent with earlier findings from studies in different parts of the UK; and they are consistent with the more general finding across the world that MS prevalence increases with increasing distance from the equator in both hemispheres. The high prevalence of MS in the North of Scotland should be recognised in funding of care for MS and for studies of disease prevention.
